# Effectiveness of Hyaluronidase on Hyaluronic Acid Degradation: An Experimental Study

**DOI:** 10.1093/asjof/ojaf108

**Published:** 2025-09-11

**Authors:** Joana Oliveira, Virgínia Santos, Joana Marques, Neusa Silva, António Mata, Mariana Brito da Cruz

## Abstract

Hyaluronidase (HYAL) is an enzyme that degrades hyaluronic acid (HA), available in both animal-derived and recombinant formulations. Currently, there is limited evidence comparing the enzyme activity of different HYALs and their effects on various HA filler formulations. The authors of this in vitro study aim to evaluate and compare the effectiveness of animal-derived vs recombinant hyaluronidase on degrading different HA filler formulations. The authors tested 4 experimental groups (*n* = 5 per group): ART FILLER Universal with animal hyaluronidase, ART FILLER Universal with recombinant hyaluronidase, ART FILLER Volume with animal hyaluronidase, and ART FILLER Volume with recombinant hyaluronidase. HA filler degradation was measured using colorimetric assay (absorbance at 585 nm) at 1, 6, 24, and 48 h of incubation. The Kruskal–Wallis test was used for statistical analysis (significance set at *P* < .05). ART FILLER Universal showed significantly greater degradation than ART FILLER Volume at the 1 h time point (*P* < .05). Recombinant hyaluronidase demonstrated consistently higher degradation activity compared with animal-derived hyaluronidase at all measurement time points, but it was only statistically significant after 1 h of incubation (*P* < .05). ART FILLER Volume with animal hyaluronidase exhibited the lowest degradation rates among all groups at 6, 24, and 48 h (*P* < .05). Based on the findings, the authors indicate that recombinant hyaluronidase is more effective than animal-derived hyaluronidase for HA filler degradation over time. ART FILLER Universal degrades more rapidly than ART FILLER Volume during the first hour of enzyme activity. The results highlight important differences in degradation kinetics between HA filler types and hyaluronidase formulations. Further studies are needed to validate these findings and explore their clinical implications.

**Level of Evidence**: 5 (Therapeutic)

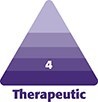

Hyaluronic acid (HA) is a glycosaminoglycan composed of 2000 to 25,0000 disaccharides of D-glucuronic acid and D-*N*-acetylglucosamine linked by β-1,4- and β-1,3-glycosidic bonds.^1^ Beyond its structural role, HA contribute to skin hydration through its remarkable water-binding capacity and protects against cellular damage from free radicals, playing a significant role in skin aging.^2,3^ For cosmetic applications, HA can be isolated from animal or bacterial sources and stabilized through cross-linking with plasticizing agents, which enhances its durability as dermal filler.^1^

Hyaluronidase (HYAL) is an endoglycosidase (specifically an endo-β-*N*-acetyl-hexosaminidase) that degrades HA by cleaving *N*-acetyl-D-glucosaminidic linkages within the HA polymer.^1,4-6^ Although the US FDA has approved HYAL for specific indications, including enhancing drug absorption, improving radiopaque agent dispersion in urography, and facilitating hypodermoclysis, its off-label applications are extensive.^7^ These include adjunct use in local anesthesia, various oncology and ophthalmology procedures, and most notably in aesthetic medicine for reversing HA filler complications.^7,8^

Meyer (1971) classified HYALs into 3 families based on their origin and mechanism of action: mammalian, microbial, and leech hyaluronidases.^9^ Mammalian HYALs, including bovine (BTH) and ovine (OTH) testicular variants, degrade HA through hydrolysis of β-1,4-glycosidic bonds. Although BTH was historically preferred, concerns over bovine spongiform encephalopathy risk have led to decreased production and increased use of OTH.^10-12^ Currently, purified mammalian HYALs are used to minimize immunological reactions.^13^

Advances in recombinant technology have enabled production of human recombinant HYAL(rHuPH20), manufactured using Chinese hamster ovary cells transfected with PH20-encoding DNA plasmid and purified to >99% purity.^10^ rHuPH20 demonstrates substantially greater specific activity (∼120,000 IU/mg) compared with animal-derived or microbial HYALs and is FDA approved for enhancing subcutaneous drug delivery (eg, morphine, insulin, and antibiotics).^10,14^ Its human origin confers greater safety, with significantly reduced risk of immunogenic reactions compared with animal-derived HYALs, which have been associated with both acute and delayed hypersensitivity responses.^10,15^

In contrast, microbial HYALs (hyaluronate lyases) degrade HA through β-elimination and hydrolysis.^4^ Produced by pathogenic bacteria including *Streptococcus* and *Clostridium* species, these enzymes facilitate tissue invasion by degrading connective tissues HA.^4,5,16^

Despite widespread clinical use, significant knowledge gaps remain regarding HYAL pharmacology. Although some studies have characterized HYAL's dose and time-dependent degradation of HA, comparative analyses of different HYAL types are lacking likely because of the absence of standardized usage guidelines.^4,17,18^ Reported dosing varies considerably (1.5-300 IU), with 1.5 to 3 IU typically required for filler nodule treatment, although refractory cases may require repeated administration or up to 10 IU which may cause allergic reactions.^19,20^

Current evidence remain limited concerning key aspects of hyaluronidase activity, including differences in enzymatic activity among HYAL types, the concentration needed to achieve equivalent HA degradation, and time course of degradation. This study aims to quantitatively compare the efficacy of animal-derived and recombinant HYALs in degrading 2 different cross-linked HA fillers using a standardized colorimetric assay.

## METHODS

We evaluated 4 experimental groups (*n* = 5 per group) consisting of ART FILLER Universal (25 mg/mL HA; FillMed, France) and ART FILLER Volume (25 mg/mL HA; FillMed), each treated with either animal hyaluronidase (1500 IU/vial; InstitutoBcn, Spain) or recombinant hyaluronidase (1500 IU/vial; pbserum, Spain). Control groups included a positive control (HA filler with + hyaluronidase without reagent) and negative controls (HA alone or HYAL alone) to validate the *N*-acetylglucosamine (NAG) detection technique.

**Figure 1.**
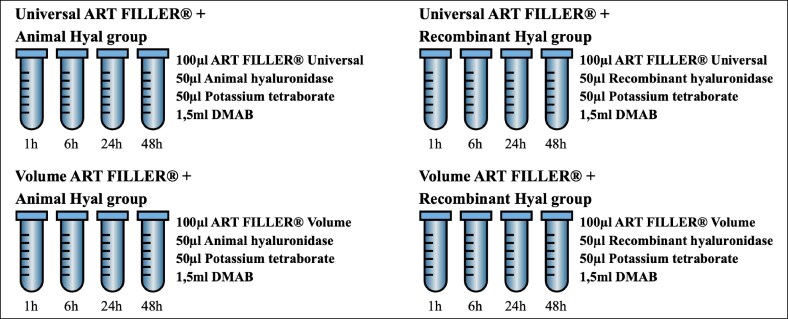
Representation of the experimental groups with the specific content present in each tube.

For experimental procedure, 0.1 mL of each HA filler were placed into Eppendorf tubes (Frilabo, Portugal), centrifugated at 1890 rpm for 5 min (to remove air bubbles and achieve a uniform matrix before initiating the enzyme digestion experiments), and preincubated at 37°C for 10 min. Enzyme reactions were initiated by adding 50 µL (75 IU) of the respective hyaluronidase to each test groups, followed by incubation at 37°C. Reactions were terminated at specified time points (1, 6, 24, and 48 h) by adding potassium tetraborate (dimethylaminobenzaldehyde, DMAB), a chromogenic reagent for NAG quantification, as illustrated in Figure 1.

Following reaction termination, samples were vortexed immediately and heated to 100°C for 3 min in a water bath. After cooling to room temperature, 1.5 mL of *p*-DMAB was added to all tubes except the negative control. Samples were then vortexed and incubated at 37°C for 20 min to allow development of the violet chromogen, whose intensity corresponds to NAG concentration. After centrifugation at 1890 rpm for 15 min, 200 µL aliquots were transferred in quintuplicate to a 96-well plate for absorbance measurements at 585 nm using a VICTOR Nivo microplate reader.

Statistical analysis was performed using IBM SPSS Statistics software (version 28; Chicago, IL, USA). Normality was assessed using Shapiro–Wilk and Kolmogorov–Smirnov tests. As the data showed non-normal distribution, nonparametric Kruskal–Wallis tests were employed, followed by pairwise comparisons. Statistical significance was set at *P*-value <.05, with results reported as mean ± standard deviation (SD).

An earlier version of this work was published online as a master's thesis in the repository of the Faculty of Dental Medicine, University of Lisbon.^21^

## RESULTS

The absorbance values of all samples were measured, with mean values (±SD) present on Table 1. The negative control group (HA alone or HYAL alone) demonstrated negligible absorbance, confirming the absence of degradation.

**Table 1.** Mean Value (±Standard Deviation) of Absorbance (A), Measured at 585 nm of the 4 Groups

Time (h)	Absorbance (A)			
	Universal ART FILLER with animal HYAL	Universal ART FILLER with recombinant HYAL	Volume ART FILLER with animal HYAL	Volume ART FILLER with recombinant HYAL
1	0.0732 ± 0.0074	0.0592 ± 0.0012	0.0526 ± 0.0012	0.0540 ± 0.0019
6	0.1284 ± 0.0014	0.1394 ± 0.0014	0.1120 ± 0.0011	0.1504 ± 0.0016
24	0.2350 ± 0.0048	0.2610 ± 0.0031	0.1984 ± 0.0025	0.2506 ± 0.0020
48	0.3032 ± 0.0021	0.3174 ± 0.0022	0.2582 ± 0.0070	0.3274 ± 0.0040

HYAL, hyaluronidase.

Exposure to hyaluronidase resulted in measurable time-dependent increases in absorbance across all samples, reflecting progressive HA degradation and subsequent NAG release. The degradation profiles revealed notable variations between experimental groups, with distinct kinetic patterns emerging throughout the observation period.

Statistical analysis using the Kruskal–Wallis test confirmed a significant difference in ART FILLER degradation by hyaluronidases across time points (*P* < .05), although post hoc pairwise comparisons indicated these differences were not uniformly distributed among all sample pairs.

**Figure 2.**
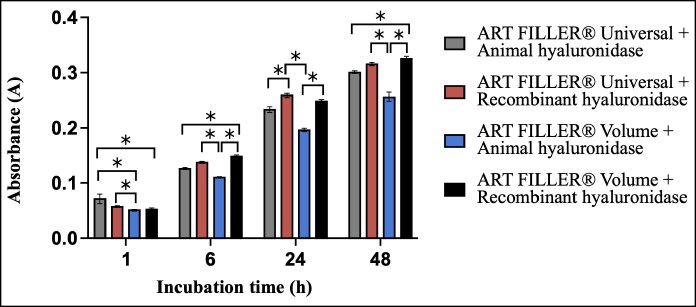
Bar graph showing the mean values ± standard deviations of absorbance that represent the quantity of *N*-acetylglucosamine release after ART FILLER Universal and ART FILLER Volume degradation by animal and recombinant hyaluronidase over time (*n* = 5). Absorbance was measured at 585 nm. *P* < .05.

At the 1-h time point, ART FILLER Universal with animal-derived hyaluronidase demonstrated the highest absorbance values. These measurements showed statistically significant differences when compared with both volume formulations, specifically, ART FILLER Volume with animal-derived hyaluronidase (*P* < .001) and ART FILLER Volume with recombinant hyaluronidase (*P* < .05). The longitudinal analysis revealed consistent patterns at later time points (6, 24, and 48 h). ART FILLER Volume degraded significantly more slowly when treated with animal-derived hyaluronidase compared with recombinant hyaluronidase. The most pronounced differences appeared when comparing ART FILLER Volume with animal-derived hyaluronidase vs ART FILLER Volume with recombinant hyaluronidase at 6 and 48 h (*P* < .001) and ART FILLER Universal with recombinant hyaluronidase at 6, 24, and 48 h (*P* < .05; Figure 2).

**Figure 3.**
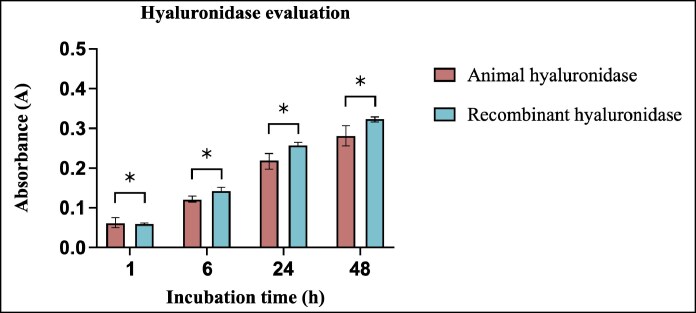
Bar graph showing the mean values ± standard deviations of absorbance that represent the quantity of *N*-acetylglucosamine release after the filler's degradation by animal and recombinant hyaluronidase over time (*n* = 5). Absorbance was measured at 585 nm. *P < .05.

Comparative analysis of enzymatic activity revealed distinct temporal patterns between hyaluronidase formulations (Figure 3). Although animal-derived hyaluronidase demonstrated greater initial efficacy in filler degradation at 1 h postincubation, this early advantage was not statistically significant (*P* < .05). However, at subsequent time points, differences emerged at 6 h (*P* < .001), 24 h (*P* < .05), and 48 h (*P* < .001). These findings indicate that although both enzymes effectively degrade HA fillers, recombinant hyaluronidase maintains consistent enzymatic activity over extended periods.

**Figure 4.**
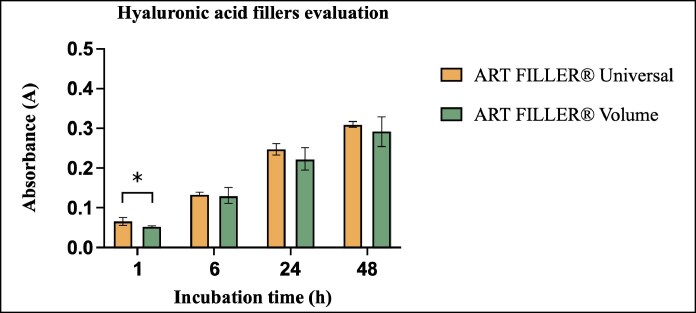
Bar graph showing the mean values ± standard deviations of absorbance that represent the quantity of *N*-acetylglucosamine release after ART FILLER Universal and ART FILLER volume degradation by hyaluronidase over time (*n* = 5). Absorbance was measured at 585 nm. *P < .05.

Comparative analysis of filler degradation revealed consistent differences between the 2 formulations (Figure 4). ART FILLER Universal demonstrated greater degradation than ART FILLER Volume at all measured time points (1, 6, 24, and 48 h). However, these differences were statistically significant only at the 1 h time point (*P* < .001), while showing consistent but nonsignificant trends at subsequent measurements.

## DISCUSSION

The increasing use of HA fillers in aesthetic procedures necessitates a thorough understanding of hyaluronidase properties and enzymatic activity to effectively manage treatment complications. Despite its clinical importance, few studies have systematically compared the degradation efficacy of different hyaluronidase formulations. Our study provides a quantitative evaluation of enzymatic activity for both animal-derived and recombinant hyaluronidases on commonly used HA fillers.

The colorimetric assay used in our study measures HA degradation through NAG release, where increased enzymatic activity procedures more intense violet chromogen formation, corresponding to higher absorbance values.^22-24^ This methodology revealed significant differences in degradation kinetics between hyaluronidase types. Although previous work by Rao et al found no differences between ovine (VITRASE) and recombinant (Hylenex) hyaluronidases across various HA fillers (Belotero, Restylane, and Juvederm), our results demonstrate recombinant hyaluronidase's superior efficacy at later time points (6, 24, and 48 h; *P* < .05).^25^ These discrepancies likely reflect differences in experimental design, including the specific hyaluronidase and filler formulations tested.

Notably, recombinant hyaluronidase exhibited delayed but more sustained enzymatic activity, suggesting it may be particularly suitable for nonurgent clinical scenarios requiring complete filler degradation. This finding aligns with literature reports of recombinant hyaluronidase higher specific activity per protein unit compared with animal-derived hyaluronidase formulations.^10^ The temporal progression of degradation activity underscores the importance of considering treatment timing when selecting hyaluronidase formulations.

Filler cross-linking density significantly influenced degradation rates.^17^ As anticipated from the manufacturer specifications, the more cross-linked ART FILLER Volume demonstrated slower degradation compared with ART FILLER Universal, with statistically significant differences at 1 h (*P* < .001). This suggests that ART FILLER Universal may be preferable when rapid degradation is clinically indicated. These observations are consistent with established principles of HA filler biochemistry, where cross-linking density inversely correlates with enzymatic susceptibility.^11,23^

Comparative analysis with Sall and Férard highlights the concentration-dependent nature of hyaluronidase activity.^26^ Although their study reported faster degradation (0.10-0.20 nm within 2 h), this likely reflects their use of substantially higher enzyme concentrations (6080 IU/mL vs ours 75 IU). This discrepancy emphasizes the need for standardized concentration-response studies to establish optimal dosing protocols. Our decision to use equivalent doses of both hyaluronidase types facilitated direct comparison, revealing distinct temporal profiles: animal-derived hyaluronidase for immediate effects vs recombinant for sustained degradation.

Although this investigation provides important insights into hyaluronidase-mediated filler degradation, some limitations should be considered when interpreting the results. The in vitro design, although carefully controlling temperature (37°C) and pH (7.4), cannot fully replicate in vivo conditions where vascular clearance and tissue interactions modulate degradation. Additionally, material constraints prevented comprehensive concentration-response analysis or Michaelis–Menten kinetics characterization. Future study should incorporate expanded concentration ranges, longer observation periods, standardized enzymatic curves, and in vivo validation.

Clinically, these findings inform evidence-based filler selection. For patients desiring temporary enhancement, fillers with ART FILLER Universal's degradation profile may be preferable, whereas those seeking longer-lasting results might benefit from more cross-linked formulations. Furthermore, understanding these degradation kinetics enables clinicians to select the most appropriate hyaluronidase type based on treatment urgency and desired extent of correction.

## CONCLUSIONS

Both animal-derived and recombinant hyaluronidases effectively degraded HA fillers in our study. However, longitudinal analysis revealed superior enzymatic activity of recombinant hyaluronidase, demonstrating significantly greater degradation efficacy at latter time points (6-48 h) compared with animal-derived hyaluronidase. Notably, ART FILLER Universal showed more rapid initial degradation than ART FILLER Volume, with statistically significant differences observed within the first hour. These findings highlight the importance of considering both hyaluronidase type and filler formulation when planning treatment reversals. Further research is needed to characterize the enzymatic kinetics of different hyaluronidase products and their interactions with various HA filler formulations.
